# From research to practice: a pilot implementation study of a falls self-efficacy tool in a community hospital

**DOI:** 10.3389/frhs.2025.1715269

**Published:** 2025-12-29

**Authors:** Yan Fang Cheryl Tan, Wei Na Lai, Shawn Leng Hsien Soh, Jiaying Ho, Rui Hong Zhao, Lian Leng Low

**Affiliations:** 1 Singhealth Community Hospitals, Singapore, Singapore; 2 Singapore Institute of Technology, Singapore, Singapore; 3 Singapore General Hospital, Singapore, Singapore

**Keywords:** falls efficacy, implementation science, patient-reported outcome measures, rehabilitation, patient safety

## Abstract

**Introduction:**

Inpatient falls are serious adverse events that contribute to functional decline and adverse outcomes. Overconfidence in mobility, and reluctance to seek assistance, are often difficult for staff to detect in the absence of a structured tool. The Multidimensional Falls Efficacy Scale (MdFES) was developed to assess patients’ confidence across fall prevention, recovery, and self-protection domains. This pilot implementation feasibility study evaluated the early adoption of the MdFES in a community hospital, using the Proctor Implementation Outcomes framework to examine patient and nurse perspectives.

**Methods:**

A mixed-methods pilot was conducted in Singapore community hospitals involving 90 patients and 32 nurses. Quantitative data were collected across multiple implementation outcomes—including acceptability, appropriateness, feasibility, cost, and fidelity—using structured questionnaires, with results reported as mean ± SD. Qualitative data from open-ended responses were thematically analysed to identify barriers and facilitators to MdFES implementation.

**Results:**

Patients reported high acceptability [Acceptability of Intervention Measure (AIM)] = 17.48 ± 2.66) and appropriateness [Intervention Appropriateness Measure (IAM)] = 17.54 ± 2.75), with 80% agreeing with their MdFES results and an average completion time of 3.12 ± 2.23 min, indicating low perceived burden. In contrast, nurses reported moderate acceptability (AIM = 12.72 ± 2.11), appropriateness (IAM = 13.19 ± 3.17), and feasibility [Feasibility of Intervention Measure (FIM)] = 13.47 ± 2.66), citing language barriers, cognitive limitations, and workflow constraints as key challenges. Fidelity was affected, with frequent rewording and translation required. Qualitative themes highlighted the need for translated versions, simplified wording, and workflow integration.

**Conclusion:**

This pilot feasibility study demonstrates that the MdFES is acceptable and meaningful to patients, while revealing modifiable feasibility challenges for nurses. These early findings provide essential insights to guide workflow adaptations, stakeholder engagement, and contextual modifications required before proceeding to a larger-scale, multi-centre implementation study.

## Introduction

Inpatient falls are serious adverse events that prolong hospitalisation, increase healthcare costs, and compromise rehabilitation outcomes (1, 2). Healthcare staff frequently perceive that patients overestimate their abilities and do not call for help before ambulating, leading to unsafe situations (3). Such judgments, however, are often based on general observation rather than structured assessments. At present, no validated tool exists within the Singapore context to objectively measure patients’ fall-related confidence or self-efficacy during inpatient rehabilitation.

Falls efficacy, an individual's confidence in preventing and managing falls, is a key psychological construct of rehabilitation that warrants greater attention (4). Measuring self-efficacy enables clinicians to identify discrepancies between perceived and actual risk, tailor interventions, and optimise patient engagement in mobility and rehabilitation activities (5). Employing a validated measurement instrument can empower patients by prompting reflection on their own confidence in preventing and managing falls, while also providing clinicians with quantifiable data to guide risk communication, patient education, and supervision strategies (6, 7).

Several patient-reported outcome measures (PROMs) exist, including the Falls Efficacy Scale (FES) developed by Tinetti et al. (8), which assesses low self-efficacy in avoiding falls during daily activities; the Activities-specific Balance Confidence (ABC) Scale developed by Powell et al. (9), measuring balance confidence in increasingly challenging tasks; and the Falls Efficacy Scale–International (FES-I) developed by Kempen et al. (10), including a short form, which evaluates concerns about falling across various activity types. While these tools measure key domains of falls efficacy, they are often lengthy, resource-intensive, and focused on specific domains such as balance confidence or concerns about falling. Assessing confidence across the entire fall event continuum: from prevention to protection to recovery, reflects the real-world trajectory of falls, identifies overlooked fears, and ensures rehabilitation strategies promote not just fall avoidance but also safe response and recovery (11).

Furthermore, despite widespread use of falls-related PROMs in research, their real-world integration into rehabilitation workflows remains underexplored, particularly in multilingual Asian contexts. Busy hospital wards require ultra-brief, comprehensible, and culturally adaptable tools that are acceptable to patients and feasible for nurses to administer within existing workflows. While FES, ABC, and FES-I are widely used in research, little is known about their implementation outcomes such as acceptability, feasibility, appropriateness, and fidelity in routine care. A recent study from Singapore highlighted barriers at the system, patient, and resource levels to implementing falls prevention programmes, including low uptake, limited sustained engagement, and poor coordination across care settings (12).

To address these gaps, the Multidimensional Falls Efficacy Scale (MdFES) developed by Shawn et al. is a brief four-item PROM that assesses patients’ confidence across key domains of fall prevention, protection, and recovery (13). Its simple structure and intuitive response format enable quick administration by nurses while minimising patient burden. Using the MdFES, findings from an unpublished study in a Singapore community hospital highlighted that over 60% of hospitalised seniors demonstrated a discrepancy between their perceived falls self-efficacy and their objectively assessed fall risk.^1^ Embedding the MdFES into clinical workflows offers an opportunity to provide rapid, actionable insights into patients’ fall-related self-efficacy and to support person-centred rehabilitation.

In Singapore, community hospitals serve as step-down care facilities, providing short-term medical and rehabilitative services to patients discharged from acute hospitals, similar to those in the United States, Europe, and Australia (14). These hospitals face the dual challenge of restoring functional independence while minimising fall risk. Local data show fall rates as high as 2.9 per 1,000 patient-days, underscoring the urgency of effective fall-prevention strategies (15).

Given the limited evidence on how falls efficacy measures can be integrated into real-world clinical workflows, there is a critical need to understand whether a brief, multidimensional tool such as the MdFES can be feasibly adopted in routine care. Falls self-efficacy influences patients’ mobility behaviour, help-seeking patterns, and fall risk, yet its assessment is seldom embedded into daily rehabilitation practice. Understanding the implementation outcomes of the MdFES is therefore essential to determine whether the tool can bridge the gap between patients’ perceived confidence and their actual fall risk, and to support safer, more person-centred care.

Accordingly, this pilot implementation feasibility study was designed to examine the acceptability, appropriateness, feasibility, and fidelity of integrating the MdFES into routine care within Singapore's community hospitals. Guided by Proctor's implementation outcomes framework, the study evaluates the perspectives of both patients who self-report their falls self-efficacy, and nurses, who administer the tool within existing workflow constraints. By identifying the barriers and facilitators that shape the use of the MdFES in practice, this pilot aims to generate implementation insights needed to optimise workflow integration, enhance usability, and inform the design of a future larger-scale, multi-centre implementation study. To our knowledge, this is the first study in Singapore to assess the early implementation of a falls self-efficacy tool in community hospital rehabilitation, addressing a significant gap in translating PROMs into routine clinical practice.

## Methodology

### Study design and setting

This study was conducted as a pilot implementation feasibility study from 1 March to 31 July 2025 in two general wards of a 382-bed community hospital in Singapore. The hospital is co-located with the country's largest tertiary acute hospital and is part of a cluster serving the eastern region. The primary aim was to evaluate the acceptability, appropriateness, feasibility, cost, and fidelity of integrating the MdFES into routine clinical workflows using the Proctor Implementation Outcomes framework, in order to determine whether a larger-scale, multi-centre implementation study would be feasible and justified (16). This framework was selected for its clearly defined, measurable constructs, which are well-suited for evaluating early-stage integration of patient-reported outcome measures (PROMs) like the MdFES in community hospital settings. Compared to broader frameworks such as the Consolidated Framework for Implementation Research (CFIR) or the Reach, Effectiveness, Adoption, Implementation, and Maintenance framework (RE-AIM), Proctor's model enables focused assessment of both patient and provider perspectives (17).

### Participants

#### Patients

All patients admitted to the study wards who could understand the MdFES (in English, Mandarin, Malay, or Tamil) were administered the scale within one week of admission as part of routine care. Patients were screened for eligibility by a study team member; inclusion required cognitive intactness and the ability to provide informed consent. Eligibility to complete the MdFES was based on the patient's ability to understand and meaningfully respond to the questionnaire, independent of their underlying medical conditions. Exclusion criteria were clinical instability, inability to communicate, or refusal to participate. Eligible patients were verbally briefed, verbal consent was documented, and each enrolled patient was assigned a unique study code to ensure confidentiality.

#### Nurses

All registered and enrolled nurses working in the two study wards were eligible and invited to participate. Participation was voluntary and anonymous, and completion of the study questionnaire was taken as implied consent.

### Sample size

For this pilot study, a pragmatic target sample size of 90 patients and 30 nurses was set to capture diverse perspectives and assess the feasibility of integrating the MdFES into routine clinical workflow. A formal power analysis was not performed, as the primary aim of this pilot was to evaluate feasibility, acceptability, and implementation processes rather than to test hypotheses. This approach is consistent with methodological guidance that feasibility studies are not typically powered for statistical significance but are instead designed to inform the parameters and requirements of a subsequent large-scale study. Procedures.

#### Training and preparation

Before study initiation, nursing staff received a 15-minute training session on MdFES administration and scoring. The scale was subsequently embedded into existing admission workflows.

#### Patients

Patient participants completed the MdFES within one week of admission as part of their routine nursing assessment. Between weeks 2 and 3 of the patient's stay, a trained study team member conducted interview-based surveys with patient participants to gather feedback on MdFES usability, relevance, and perceived burden.

#### Nurses

Nurses were invited to complete a self-administered questionnaire via a secure online data collection platform. The questionnaire evaluated their experiences with MdFES integration, including usability, workflow fit, feasibility, and resource needs.

### Measures

A combination of validated scales and study-specific questionnaires to evaluate both patient and nurse perspectives on the MdFES, as well as to assess key implementation outcomes was employed.

#### MdFES

The MdFES (Annex 1) is a brief, 4-item instrument that assesses an individual's confidence in preventing and managing falls across four domains: balance confidence (Item 1), balance recovery confidence (Item 2), safe landing confidence (Item 3), and post-fall recovery confidence (Item 4). Each item is rated on a 5-point scale from 0 (not at all confident) to 4 (completely confident), yielding a total score ranging from 0 to 16, with higher scores indicating greater falls efficacy. Items 1 and 2 constitute the falls-prevention subscale, while Items 3 and 4 form the falls-management subscale. The MdFES has demonstrated good internal consistency (Cronbach's alpha = 0.84) and a two-factor structure explaining 65.4% of the total variance in its initial validation study. These properties support its use as a concise, multidimensional measure of falls efficacy. Permission to use the MdFES was obtained from the original author prior to data collection.

#### Implementation outcome measures

Three validated measures were incorporated: the Acceptability of Intervention Measure (AIM), Intervention Appropriateness Measure (IAM), and Feasibility of Intervention Measure (FIM) to aid in the development of our questionnaires (18). Each scale ranges from 0 to 20, with higher scores indicating better implementation outcomes. For interpretation, scores ≥16 were considered high, scores between 12 and 15 indicated moderate acceptability, appropriateness, or feasibility, and scores ≤11 reflected low ratings, suggesting that substantial modifications might be required to improve integration. These scales are available instruments in the public domain and did not require additional permissions.

#### Patient questionnaire

The patient questionnaire evaluated their willingness to engage with the MdFES (adoption), ease of understanding and perceived relevance (acceptability), usefulness of the tool within their rehabilitation context (appropriateness), and the time and effort required to complete it (cost).

#### Nurse questionnaire

The nurse questionnaire assessed a broader set of implementation outcomes. These included the extent of MdFES use in routine care (adoption), perceived usability and integration into workflows (acceptability), alignment of the tool with patient care needs (appropriateness), and time and resource demands (cost). In addition, we explored feasibility, defined as the practicality of embedding MdFES within existing workflows, and fidelity, referring to the consistency of administering the tool as intended.

Open-ended questions were also included to capture qualitative insights. Patients were invited to comment on the relevance and usability of the MdFES, whereas nurses reflected on barriers and facilitators to adoption and whether use of the tool influenced their management strategies for patients.

This approach enabled a comprehensive evaluation of both patient and nurse perspectives on the acceptability, appropriateness, feasibility, and broader integration of the MdFES, thereby generating practical insights to guide future scale-up in similar rehabilitation settings.

### Statistical analysis

A stepwise analytical approach was used to evaluate both quantitative and qualitative data in this mixed-methods pilot implementation study. Quantitative implementation outcomes were first summarised using descriptive statistics. Mean scores and standard deviations were calculated for the AIM, IAM, and FIM, as well as for reported completion times. Categorical responses, including awareness of the MdFES, perceived effort, and fidelity indicators such as rewording or translation of items, were summarised as frequencies and percentages. Completion rates were also calculated to determine the proportion of eligible patients who were able to complete the MdFES during the study period.

Implementation outcomes for patients and nurses were then examined descriptively to identify similarities and differences in their perceptions of the acceptability, appropriateness, and feasibility of MdFES use in routine clinical workflows. Interpretation of AIM, IAM, and FIM scores followed established thresholds, with scores ≥16 indicating high ratings, 12–15 reflecting moderate ratings, and ≤11 suggesting low ratings.

Qualitative data from open-ended responses were analysed using an inductive, qualitative descriptive approach. Narrative comments were reviewed in full, and recurring ideas were identified and grouped together. These groups were then refined into broader themes that reflected participants’ perspectives, including perceived value, barriers to administration, facilitators, role alignment, and perceived impact on patient care.

### Ethical considerations

This study was approved by the SingHealth Centralised Institutional Review Board (CIRB) (Ref: 2024-4350). Informed consent procedures were followed for all participants, and confidentiality was maintained.

## Results

### Quantitative findings

**Figure 1:**
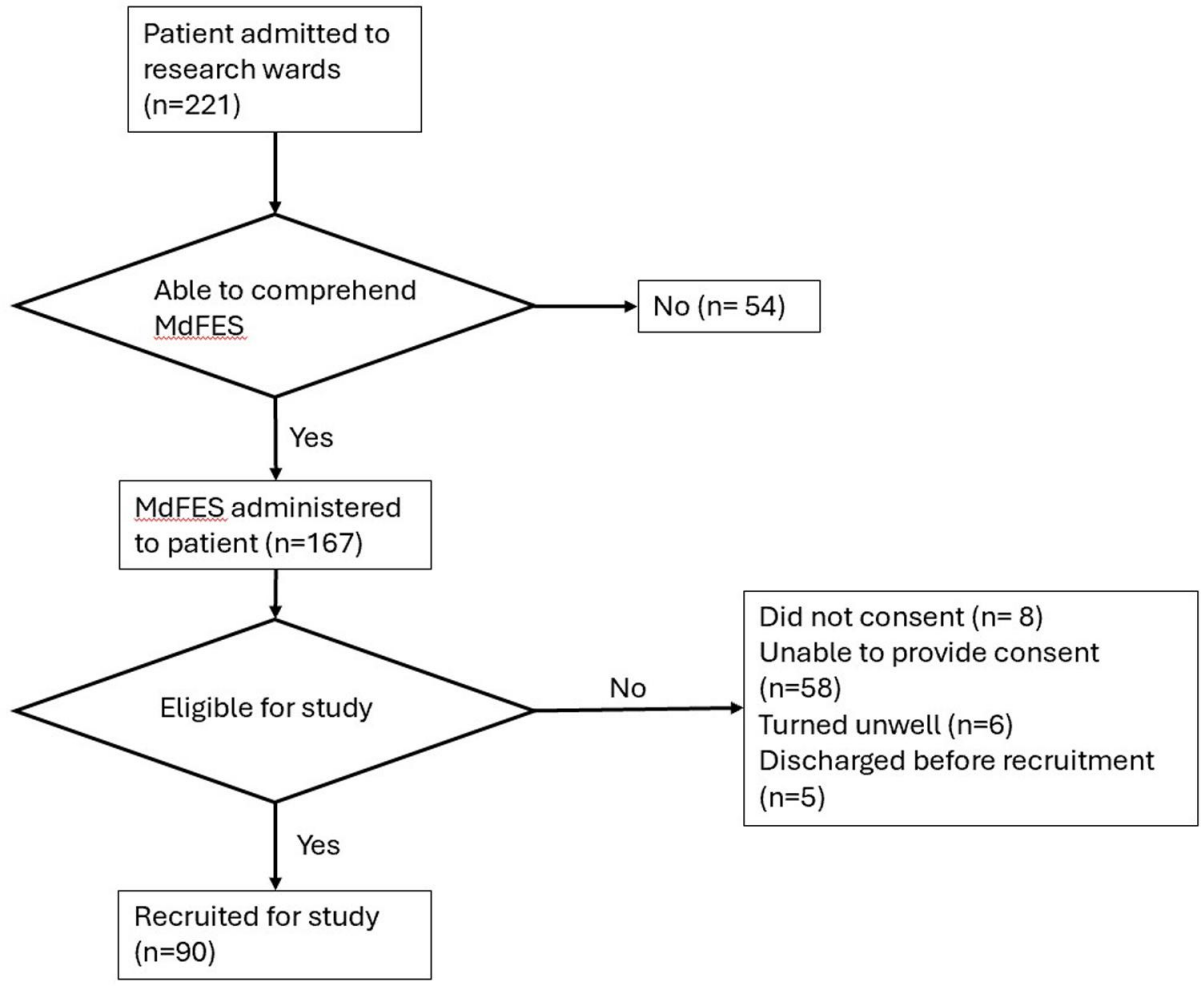
Study recruitment and data collection flow for patient participants. MdFES, multidimensional falls efficacy scale.

As shown in Figure 1, the MdFES was administered to 167 of 221 admitted patients (75.6%). 90 (53.1%) completed the MdFES, and 77 (46.1%) could not complete the MdFES due to comprehension, lack of consent, or clinical reasons.

Quantitative implementation outcomes were presented in Table 1. Awareness of the MdFES was high, with 95.6% of patients and all nurses being aware of the tool.

**Table 1:** Quantitative implementation outcomes of the MdFES reported by participants.

Implementation outcome	Measure		Patients (*n* = 90)	Nurses (*n* = 32)
Adoption	Aware of MdFES		86 (95.6%)	32 (100%)
	Intention to use (stages of change)	Precontemplation	–	7 (21.88%)
		Preparation	–	1 (3.13%)
		Contemplation	–	8 (25.00%)
		Action	–	11 (34.38%)
		Maintenance	–	5 (15.63%)
Acceptability	Agreement with scale results	Completely disagree	3 (3.33%)	1 (3.13%)
		Disagree	7 (7.78%)	–
		Neutral	8 (8.89%)	22 (68.75%)
		Agree	22 (24.44%)	9 (28.13%)
		Completely agree	50 (55.56%)	–
	Acceptabilty of intervention measure (0–20): mean		17.48 (SD 2.66)	12.72 (SD 2.11)
Appropriateness	Understanding of MdFES questions	Does not understand completely	2 (2.22%)	–
		Does not understand some	6 (6.67%)	–
		Neutral	8 (8.89%)	–
		Understands most	16 (17.78%)	–
		Understands completely	58 (64.44%)	–
	Intervention appropriateness measure (0–20): mean		17.54 (SD 2.75)	13.19 (SD 3.17)
Feasibility	Feasibility of intervention measure (0–20): mean		–	–
Fidelity	Frequency of rewording MdFES items for patient comprehension	Never	–	1 (3.13%)
		Rarely	–	4 (12.50%)
		Sometimes	–	13 (40.63%)
		Often	–	12 (37.50%)
		Always	–	2 (6.25%)
	Frequency of MdFES translation for patient comprehension	Never	–	1 (3.13%)
		Rarely	–	5 (15.63%)
		Sometimes	–	12 (37.50%)
		Often	–	11 (34.38%)
		Always	–	3 (9.38%)
Cost	Effort to complete the scale	Huge effort	2 (2.22%)	4 (12.50%)
		Some effort	11 (12.22%)	12 (37.50%)
		Neutral	6 (6.67%)	4 (12.50%)
		A little effort	12 (13.33%)	11 (34.38%)
		No effort at all	59 (65.56%)	1 (3.13%)
	Time taken (mins)		3.12 (SD 2.23)	7.13 (SD 6.10)
	Perceived interference of MdFES administration with other priorities	Yes	–	10 (31.25%)
		No	–	22 (68.75%)
	Self-reported confidence administering MdFES	^a^Neutral	–	15 (46.88%)
		Confident	–	17 (53.13%)
Others	Perceived usefulness of MdFES in identifying high fall-risk patients	Completely not useful	–	1 (3.13%)
		Not useful	–	4 (12.50%)
		Neutral	–	11 (34.38%)
		Useful	–	15 (46.88%)
		Very useful	–	1 (3.13%)
	Potential of integrating MdFES into nursing assessment	Yes		25 (78.13%)
		No		7 (21.88%)

a There were zero responses for completely not confident, not confident, and completely confident.

Patients generally rated the tool positively, reporting high acceptability (mean AIM 17.48, SD 2.66) and appropriateness (mean IAM 17.54, SD 2.75), with 80% agreeing with their results and 82.2% understanding most or all items.

Nurses expressed more moderate views, with moderate acceptability (mean AIM 12.72, SD 2.11; 68.8% neutral), appropriateness (mean IAM 13.19, SD 3.17) and feasibility (mean FIM 13.47, SD 2.66).

There was longer reported administration times for nurses (7.13 min) compared to patients (3.12 min).

Fidelity challenges were evident, with most nurses frequently rephrasing (40.6% sometimes, 37.5% often) or translating items (37.5% sometimes, 34.4% often). Perceived effort was low for patients (65.6% “no effort at all”), but higher for nurses, with 37.5% reporting “some effort” and 31.3% noting interference with other priorities.

50% of nurses found the tool useful for identifying high-risk patients and over three-quarters (78.1%) supported its integration into clinical assessments.

### Qualitative findings

To complement the quantitative outcomes, qualitative feedback from patients and nurses were presented in Table 2, providing insights into the perceived value, barriers, and facilitators of MdFES implementation.

**Table 2:** Perceptions of MdFES implementation reported by participants.

Theme	Stakeholder	Subtheme	Description
Perceived value & relevance	Nurses	Usefulness for clinical insight	MdFES helped gauge patients’ confidence and overestimation of ability, especially at admission.
	Nurses	Overlap with existing assessments	Seen as duplicating other fall risk tools, limiting added value.
	Patients	Integration with rehab & education	Wanted results to guide therapy and education.
Barriers to administration	Nurses	Clarity and wording of items	Questions seen as too broad, difficult, or confusing.
	Nurses	Language and literacy barriers	Frequent need for translation, rephrasing, simplification.
	Nurses	Cognitive suitability	Patients with dementia, confusion, or poor capacity could not reliably answer.
	Nurses	Time & workflow burden	Time constraints, documentation burden, translation challenges.
	Patients	Timing of administration	Suggested administering later or multiple times.
	Patients	Clarity & relevance of questions	Questions too general; suggested adding/rephrasing.
Role alignment and responsibility	Nurses	Perceived role alignment	Felt more relevant for therapists than nurses.
	Patients	Administration process & roles	Suggested physiotherapists may administer; rapport affects quality.
Facilitators & enablers	Nurses	Facilitators for use	Embedding into workflow and using visuals eased administration.
	Patients	Contextual understanding of falls	Highlighted need for contextualized scenarios and environmental factors.
Impact on care	Nurses	Impact on management	Results influenced fall prevention strategies and attention to high-risk patients.

Theme 1: Perceived Value & Relevance

Both patients and nurses recognised the potential value of the MdFES, though their perceptions of its relevance differed.

Nurses described the tool as helpful for gauging patients’ confidence and identifying overestimation of ability, particularly at admission.

“It gives us a gauge on how patient feels… we are able to gauge and adjust accordingly.” – Nurse 1

At the same time, some questioned its added value, noting overlap with other assessments.

“The Fall risk assessment is already enough to assess patient.” – Nurse 3

Patients, in contrast, emphasised the MdFES's role in supporting rehabilitation and recovery. Several highlighted its potential to guide therapy and education on fall prevention and recovery.

“Education on fall recovery may help patients improve fall efficacy.” – Patient 39

Theme 2: Barriers to Administration

Nurses identified several challenges that hindered the routine use of the MdFES. Time pressures and competing clinical demands were frequently cited.

“*Time consuming*.” – Nurse 12

Others highlighted difficulties related to language and literacy, noting the need for translation and simplification when communicating with patients.

“Language barrier (if staff on duty foreigner and the patient speaks local language).” – Nurse 16

Cognitive limitations among patients also posed obstacles when nurses assisted in administering the MdFES.

“Applicable only for those cognitively intact who can answer questions.” – Nurse 23

Patients raised related concerns, particularly around the timing and clarity of administration. Several suggested that the scale might be more meaningful if administered later during admission, once they had a clearer understanding of their condition.

“Possible to administer at a later date due to lack of understanding during admission.” – Patient 6

Others felt that the items were overly general, recommending greater specificity

“Phrasing of questions: elaborate what type of fall e.g., from what height?” – Patient 50

Theme 3: Role Alignment and Responsibility

Nurses felt the tool aligned more closely with the responsibilities of therapists rather than nursing workflows.

“It's most relevant to therapist side.” – Nurse 13

Echoing nurses’ views on role alignment, some also suggested that physiotherapists, rather than nurses, might be better positioned to administer the tool given their rehabilitation focus and rapport with patients:

“Nurses are busy so physiotherapist should administer.” – Patient 55

Theme 4: Facilitators & Enablers

Despite the barriers reported, both patients and nurses identified factors that could support the use of the MdFES in routine practice. Nurses noted that embedding the tool into existing workflows and pairing it with visual aids helped streamline administration and reduce patient confusion.

“Part of admission and discharge workflow during pilot study.” – Nurse 6

Patients, meanwhile, emphasised the importance of contextualising the questions with real-life scenarios and environmental factors, which they felt would make the tool more relevant to their daily experiences

“Include section on potential hazards in daily life as part of fall prevention” Patient 19

Theme 5: Impact on Care

Both patients and nurses highlighted the potential of the MdFES to influence clinical decision-making and rehabilitation strategies. Nurses reported that the tool could draw attention to patients at higher risk of falls, prompting closer monitoring and targeted preventive measures.

“More attention can be given to high fall risk patients” – Nurse 14

Patients similarly recognised its potential role in shaping their rehabilitation journey, particularly through education on recovery strategies after a fall.

“Education on fall recovery may help patients improve fall efficacy” – Patient 39

## Discussion

This study examined the pilot adoption of the MdFES within a community hospital in Singapore, evaluating both patient and nurse perspectives through the lens of Proctor's implementation outcomes framework (16). Findings demonstrated that the MdFES was highly acceptable and appropriate for patients. However, moderate feasibility and integration challenges were identified from the nurses’ perspectives. These insights provide important considerations for embedding patient-reported outcome measures (PROMs) like the MdFES into real-world rehabilitation workflows.

A key strength of the MdFES lies in its potential to address a critical contributor to inpatient falls: patients’ confidence in their abilities and reluctance to seek help before mobilising. As described in the introduction, this behaviour is difficult for staff to detect in the absence of a structured tool. Our results showed that the MdFES was meaningful to patients, with most agreeing with its results and perceiving it as relevant to their rehabilitation. By making patients explicitly aware of their own confidence levels, the MdFES may promote self-reflection and empower patients to recognise when assistance is required, thereby complementing existing fall-risk assessments that focus primarily on objective risk. This aligns with prior evidence showing that PROMs can enhance patient engagement when they resonate with lived experiences (19–21). Patients emphasised the value of timing and education, noting that the MdFES could be more meaningful later in admission and that its results could guide fall-recovery discussions. Moreover, some viewed the MdFES as a springboard for fall-prevention education, emphasising its dual potential as both an assessment and educational tool.

In contrast, nurses reported only moderate acceptability and appropriateness, citing concerns around workflow disruption, duplication with existing fall-risk assessments, and additional resource demands. Such reservations are consistent with prior PROM implementation studies, where frontline staff questioned whether the benefits of PROMs justified the added effort (22). Clarifying the MdFES's unique contribution: capturing falls-related self-efficacy, which independently predicts falls and functional outcomes rather than duplicating risk assessments, may help to improve buy-in to reduce inpatient falls (23, 24).

Implementation challenges were compounded by language diversity, cognitive limitations, and health literacy barriers, consistent with prior findings in multicultural healthcare settings. Nurses frequently reworded or translated MdFES items to ensure comprehension, underscoring the need for culturally and cognitively adapted tools. These findings are consistent with prior research showing that PROM feasibility improves when instruments are translated, simplified, and supported by visual aids, particularly in multilingual Asian contexts (25, 26). Such adaptations ensure that PROMs remain inclusive and patient-centred, allowing equitable assessment across diverse rehabilitation populations.

The question of role alignment also emerged. While nurses supported integration of the MdFES into admission or discharge workflows, they expressed concern regarding competing clinical priorities, with some suggesting that physiotherapists might be better suited to administer the tool. Patients echoed this view, noting that therapists often build stronger rapport and could link MdFES results more directly to rehabilitation strategies. Such perspectives highlight the importance of tailoring implementation not only to workflows but also to professional roles, consistent with implementation science principles of contextual adaptation and stakeholder engagement (27).

Future implementation may be facilitated by digital integration. Embedding the MdFES into electronic health records could reduce documentation burden, improve data accessibility, and minimise duplication with existing assessments. Combining role alignment, workflow tailoring, and technological support could enhance feasibility and sustainability in routine practice.

This study has several limitations. First, the small sample size limited statistical power and precluded subgroup analyses by language or cognitive status. Second, reliance on self-reported measures may have introduced response and social desirability bias, particularly among patients. Third, physiotherapists, whom both patients and nurses identified as relevant stakeholders, were not included in this pilot. Finally, fidelity and longitudinal changes in falls self-efficacy were not assessed, limiting evaluation of the MdFES's responsiveness over time.

Further research should involve multisite studies with larger and more diverse samples to validate findings, examine adapted versions of the MdFES for multilingual and cognitively impaired populations, and explore integration into digital platforms. Longitudinal studies are also warranted to assess whether MdFES scores track meaningful changes in falls self-efficacy and predict rehabilitation outcomes. Inclusion of physiotherapists and other allied health professionals in future evaluations will also be important for refining role allocation and optimising workflow integration.

## Conclusion

This pilot implementation study demonstrates that the MdFES is acceptable and meaningful to patients, while revealing modifiable feasibility challenges from the nursing perspective. These early findings provide important insights into workflow requirements, role alignment, and contextual adaptations needed for successful integration. By identifying barriers and facilitators at the point of care, this pilot offers foundational guidance for planning a future larger-scale, multi-centre implementation study of the MdFES in routine rehabilitation settings.

## Data Availability

The original contributions presented in the study are included in the article/Supplementary Material, further inquiries can be directed to the corresponding author.

## Footnotes

1 Tan YFC, Soh LHS, Chua HPW, Xia JWO, Ho JY, Ong PH, et al. Mind versus Body: discrepancy between falls-related perceived self-efficacy and fall risks among hospitalised seniors in a Singapore community hospital (2024).

## Annex 1

### The multidimensional falls efficacy (MdFES) scale

The MdFES is a quick and simple method to rate your confidence in your ability to prevent and manage falls.

Please rate your degree of confidence by giving us a percentage below

0 - Not at all confident (0% confident)

1 - Slightly confident (25% confident)

2 - Somewhat confident (50% confident)

3 - Quite confident (75% confident)

4 - Extremely confident (100% confident)

**Table:**

S/N	Item	Score
1	*How confident are you to walk steadily?*	
2	*How confident are you to stop yourself from falling when you lose balance?*	
3	*How confident are you to protect yourself if you fall?*	
4	*How confident are you in getting up (from the ground) after a fall?*	
